# Exposure of trees to drought-induced die-off is defined by a common climatic threshold across different vegetation types

**DOI:** 10.1002/ece3.1008

**Published:** 2014-03-06

**Authors:** Patrick J Mitchell, Anthony P O'Grady, Keith R Hayes, Elizabeth A Pinkard

**Affiliations:** 1 CSIRO Ecosystem Sciences and Climate Adaptation FlagshipCollege Rd, Sandy Bay, TAS, Australia; 2 CSIRO Computational Informatics, Castray EsplanadeHobart, TAS, Australia

**Keywords:** Drought, extreme events, forest die-off, heat waves, tree mortality

## Abstract

Increases in drought and temperature stress in forest and woodland ecosystems are thought to be responsible for the rise in episodic mortality events observed globally. However, key climatic drivers common to mortality events and the impacts of future extreme droughts on tree survival have not been evaluated. Here, we characterize climatic drivers associated with documented tree die-off events across Australia using standardized climatic indices to represent the key dimensions of drought stress for a range of vegetation types. We identify a common probabilistic threshold associated with an increased risk of die-off across all the sites that we examined. We show that observed die-off events occur when water deficits and maximum temperatures are high and exist outside 98% of the observed range in drought intensity; this threshold was evident at all sites regardless of vegetation type and climate. The observed die-off events also coincided with at least one heat wave (three consecutive days above the 90th percentile for maximum temperature), emphasizing a pivotal role of heat stress in amplifying tree die-off and mortality processes. The joint drought intensity and maximum temperature distributions were modeled for each site to describe the co-occurrence of both hot and dry conditions and evaluate future shifts in climatic thresholds associated with the die-off events. Under a relatively dry and moderate warming scenario, the frequency of droughts capable of inducing significant tree die-off across Australia could increase from 1 in 24 years to 1 in 15 years by 2050, accompanied by a doubling in the occurrence of associated heat waves. By defining commonalities in drought conditions capable of inducing tree die-off, we show a strong interactive effect of water and high temperature stress and provide a consistent approach for assessing changes in the exposure of ecosystems to extreme drought events.

## Introduction

Drought is a pervasive feature of forest ecosystems that constrains primary productivity (Zhao and Running 2010) and, during extreme conditions, can induce large-scale dieback (loss of above-ground tissues) or mortality episodes (Breshears et al. 2005). A global surge in the study of drought-related impacts on forests has helped to document an increasing number of mortality events in the literature and implicate temperature increases as amplifying moisture deficit, heat stress, and the impacts of biotic agents on tree species (Allen et al. 2010; Toomey et al. 2011; Williams et al. 2013). In Australia, forest and woodland ecosystems are strongly influenced by large climatic variability and recurring drought events. Recently, these drought patterns have been affected by systematic shifts in precipitation and rising temperature. Indeed, recent drought-related forest die-off events observed in southwestern Australia (Matusick et al. 2013) were accompanied by increases in mean temperatures and the incidence of short periods (>2 days) of temperature extremes or heat waves likely to induce acute heat stress preceded by a long-term (∼40 years) decline in mean annual rainfall (Fig. S1). Drought-induced forest die-off could either represent episodic disturbances within an existing climate regime or be indicative of a climate shift. While episodic disturbances are likely to facilitate stability of forest structure and function over the long-term (Lloret et al. 2012), climatic shifts will induce significant changes in existing forest dynamics and the potential for state changes (Rietkerk et al. 2004) The challenge in disentangling which of these two processes dominates lies in defining the frequency, intensity, duration, and trajectory of climate extremes capable of inducing die-off so that future scenarios may be evaluated based on the likelihood of drought events.

Drought is defined as a prolonged and exceptional deficit between water supply and demand, in our case at the tree or stand level. It can be characterized by both its duration and intensity and is often associated with periods of above average temperatures and heat waves (Déry and Wood 2005). Species and communities may be differentially impacted by drought (Fensham and Fairfax 2007; Koepke et al. 2010; Mitchell et al. 2008), being dependent on the primary meteorological drivers, and secondary factors such as the presence of biotic agents and conditioning factors that determine site and species sensitivity to drought (Mitchell et al. 2013a). The mechanisms by which drought intensity and duration mediate species sensitivity through the physiological processes controlling plant carbon and water balance remain unresolved (McDowell et al. 2008; Sala 2009). Recent conceptual models have implicated two interrelated physiological mechanisms: hydraulic failure (desiccation of water conducting tissues within the plant) and carbon starvation (depletion of available carbohydrates and failure to maintain defenses against biotic agents) (McDowell et al. 2008, 2011) as being the primary mechanisms through which trees may succumb to drought stress. Within this framework, the relative importance of these two underlying physiological processes is intimately linked to the key attributes of the drought regime; intensity, duration, and frequency and how biotic and abiotic factors modulate plant water availability through time (Anderegg et al. in press).

Rising temperatures and the associated increase in the frequency of heat waves further increases the risk of mortality by altering plant water and carbon balances or by increasing the presence and activity of biotic agents, regardless of changes in precipitation regimes. Increasing evaporative demand will intensify drought conditions in the root zone and/or plant canopy and accelerate dehydration resulting from irreversible embolism of the xylem (Brodribb and Cochard 2009; Sperry et al. 2002). Increases in plant growth, tree size and possibly leaf area index associated with elevated temperature and CO_2_ concentrations under favorable conditions could intensify intertree competition (Warren et al. 2011) and increase forest vulnerability to mortality during sudden or protracted periods of drought (Duan et al. 2013; Levanič et al. 2011). Rising temperatures can also increase the activity of biotic agents by weakening plant defenses (Boyer 1995) or provide suitable conditions for new pest species (Kurz et al. 2008), thereby increasing the severity of plant stress during drought. The impacts of elevated temperatures on plant functioning represent a critical dimension of drought risk, and the potential for interactive effects operating in concert with the consequences of water deficit therefore need to be considered when characterizing drought.

The role of climate extremes may be critical in shaping future species and ecosystem dynamics (Zimmermann et al. 2009), but because of the sporadic and unpredictable nature of these extreme events, they remain difficult to document and monitor through time and space. The manner in which drought is defined and described in relation to ecosystem processes tends to be context specific (Smith 2011) and restricts our ability to compare the climatic conditions associated with reported episodic die-off events from different regions and climates. Recent evidence from global meta-analyses of hydraulic safety among woody species from a range of climates suggest that many species' water transport systems have evolved to operate very close to the limits imposed by their environment (Choat et al. 2012). This physiological evidence and the occurrence of episodic forest die-off events across a wide range of forest types (Allen and Breshears 1998; Keith et al. 2012) would suggest that climate thresholds on plant processes such as mortality may be reached at similar extremes relative to the long-term climatic distribution of relevant climatic parameters.

The documented occurrences of tree die-off across different ecosystems provide *a priori* evidence that critical thresholds have been surpassed with respect to plant functioning and health. However, the lack of consistent ecological information available from historic events limits the applicability of predictive modeling techniques in many cases. An alternative approach is to use the primary drivers, that is, the climatology to build a consistent profile of the drought responsible for inducing known occurrences of tree die-off. This is akin to environmental envelope techniques used to describe presence-only records of species (Pearce and Boyce 2006). We sought to develop a suitable approach that represents key climate drivers of these events within a probabilistic framework such so that drought attributes are described in terms of deviations from long-term mean conditions irrespective of underlying differences in climatology. The approach also defines the mean intensity, duration, and frequency of discrete events given their relevance to plant functioning and the underlying mechanisms responsible for tree mortality. This approach allowed us to address the following questions: (1) What are the key climatic thresholds of documented events based on commonalities in relevant indices among different sites? (2) What is the extent to which high temperature co-occurs with high levels of water deficit? (3) What are the projected changes in the frequency, intensity, and duration of extreme drought based on a relatively dry and moderate warming scenario for Australia?

## Materials and Methods

Australia is a predominately water-limited environment, and ecosystem productivity is tightly coupled to precipitation (Adams 1995). Here, we used Australia as a case study to investigate the predisposing climatic conditions associated with documented die-off events identified from a survey of published data. The majority of global circulation models (GCM) predict declines in precipitation across much of Australia coupled with increases in mean annual temperatures and exceptionally hot periods (Hennessy et al. 2008; Lucas et al. 2007). Our probabilistic approach uses the standardized precipitation evapotranspiration index (SPEI) and a similar maximum temperature index (MTI; see below) to define those drought and temperature stress thresholds (based on univariate and joint distributions) and drought attributes (intensity, duration, and frequency) associated with historic die-off events across a range of forest and woodland communities. We then evaluate the likelihood of future conditions surpassing these thresholds using a moderate warming scenario with GCM-derived climate data (2011–2050) and finally conduct a sensitivity analysis based on observed climate (1961–2010) scaled to match future patterns in temperature and precipitation at 2050.

### Drought die-off events

We conducted a literature search using various online databases to identify incidences of tree mortality and dieback that were primarily attributed to drought or where there was evidence that drought was the trigger for tree death or canopy collapse. Die-off events were identified from on-ground measurements of stand health that showed evidence of mortality or canopy collapse across a range of size classes, followed by significant increases in mortality during or directly after the event. For all events, drought damage or mortality was estimated at >5% of the individuals in relatively mature-aged stands, using a range of sampling strategies. Two sites (Canberra and Hobart) had multiple drought events, and their thresholds were assessed for each event, that is, 15 sites and 17 events in total. Data on the severity and extent of the reported tree die-offs ranged from long-term plot inventory measurements that had been repeatedly sampled through time (Cunningham and Walker 1973) to opportunistic observations of die-off (Pook et al. 1966). The 15 documented sites included in the analyses ranged from wet sclerophyll forest, to open woodland and shrub lands that were generally dominated by *Eucalyptus* spp. or *Acacia spp*. (mean annual precipitation 240–1161 mm) and covered many different biomes across Australia (Table S2). Biotic agents such as wood-boring insects were found to be present in about half of the die-off events reported (Table S2).

### Climate analysis

Daily meteorological data were extracted from the meteorological station closest to each of the 15 sites (<30 km away) using patched point datasets – observed station data where missing or suspect values are “patched” with interpolated data (http://www.longpaddock.qld.gov.au/silo/) (Jeffrey et al. 2001). Among all 15 stations, an average of 20% and 50% of the rainfall and temperature data, respectively, were “patched” following the methods outlined by Jeffrey et al. (2001). Monthly climatic water deficits (1891–2012) were calculated using the SPEI (Vicente-Serrano et al. 2009). The SPEI is a simple climatic water balance based on the difference between precipitation and potential evapotranspiration (calculated from Thornthwaite's equation), that is, de-seasonalized and subsequently standardized, so that drought conditions at different locations, over a variety of predefined time scales, can be represented as probabilities and presented in a comparable fashion. The formulation of SPEI helps to remove the seasonal autocorrelation in the water balance time series by fitting a three parameter log-logistic distribution separately to each calendar month. The standardization technique allows for direct comparison of SPEI values among sites and transforms the fitted data to standard normal, that is, mean is approximately zero and the standard deviation is one (Vicente-Serrano et al. 2009). Rather than adopting the full physical formulation of evapotranspiration provided by models such as Penman-Monteith, the Thornthwaite formulation of potential evapotranspiration was used to estimate monthly climatic water deficit due to inherent limitations in existing and future datasets (particularly the availability of daily relative humidity and net radiation data at all sites). We tested whether the Penman-Monteith equation (FAO 56, Allen et al. 1998) produced significantly different patterns in SPEI among the 15 sites over the observed 121 year time series. The SPEI values from the two methods were highly correlated (*R*^2^ = 0.95–0.99), and their slopes were not significantly different from one. Furthermore, both simulations exhibited similar minimum SPEI values during the documented die-off events. We used a SPEI calculated for a 6-month analysis window for the identification and computation of drought events and their attributes using the methods described in Vincente-Serrano et al., (2009). The 24 and 48 months SPEI values were also calculated to assess whether there was a longer-term signal in drought conditions prevailing at the start of the documented die-off events. By construction, the SPEI has a standard normal distribution; hence, negative values represent periods of net negative water balance, and the probability of exceeding values of −1 and −2 is approximately 0.16 and 0.02, respectively.

We developed a maximum temperature index (MTI) based on the monthly 90th percentile of daily maximum temperature. The MTI provides a monthly estimate of high temperature stress and is similarly standardized to allow for comparison across different climates. Specifically, we recorded the daily maximum air temperature from the observed climatic record (1891–2012), extracting from this the 90th percentile for each month. We de-seasonalized this by fitting a linear model with sinusoidal covariates on a 12-monthly period. After checking for trend, we fitted a three parameter log-logistic distribution to the residuals using the method of L moments, double-checked parameter estimates with maximum-likelihood estimates, and subsequently transformed the probability distribution of the log-logistic quantiles to a standard normal distribution. The process of deseasonalizing the data, fitting log-logistic distributions to the residuals, and then transforming these to standard normal was identical to the procedure used in the construction of the SPEI (Vicente-Serrano et al. 2009).

The joint distributions of SPEI and MTI were fitted using three copulas: Gaussian, Student-*t* and Frank. Copulas were used because they are capable of capturing linear and nonlinear forms of dependence between their marginal inputs and are therefore a more powerful tool for dependence analysis than say correlation coefficients. A Gaussian copula fitted to marginal Gaussian distributions (as is the case here) returns the well-known multivariate Gaussian distribution, characterized by the linear correlation matrix Ω. We used this to represent the “null hypothesis” of simple linear dependence between SPEI and MTI. The Student-*t* copula is parameterized by the linear correlation matrix Ω and a degrees-of-freedom parameter *ν*. This copula, like the Gaussian, is symmetrical, and the Gaussian copula is a special case of the Student-*t* as *ν* approaches infinity. Importantly, however, at low degrees of freedom the Student-*t* copula allows for tail dependence – that is a greater degree of dependence between the marginal covariates as their values become much higher, or much lower, than their means. The potential for tail dependence in this context is important because this would indicate a greater probability of vegetation experiencing both high water deficit and high temperature stress than what would occur under a normal (Gaussian) distribution. The Frank copula is also parameterized by a single dependence parameter and serves as an alternative to the Gaussian, particularly as it lacks tail dependence and when fitted to standard normal marginals displays greater dependence around the marginal means. In other words, this implies that the probability of conditions of high water deficit and temperatures are less than what would be expected under a normal distribution. All copulas were fitted using the “copula” library in the R program (v2.11.1, R Foundation for Statistical Computing, Vienna, Austria) with maximum-likelihood parameter estimation. The final copula for each site was chosen using a simple likelihood ratio test (see Table S3).

Drought events were identified from the time series of SPEI using a 6-month timescale. We used this timescale because meteorological drought can be as much as 6 months out of phase with soil water deficit (Mpelasoka and Chiew 2009) and droughts shorter than 6 months are unlikely to place severe stress on the predominately sclerophyllous vegetation of Australia. A drought event was defined as occurring whenever the SPEI values were ≤−1 following (McKee et al. 1993). The drought commenced when the SPEI value became less than zero and ended when the values had recovered back to zero or higher. Drought duration was calculated by summing the months during this period, and drought intensity was taken as the mean SPEI over the drought period (see Fig. S1 for an example). The relationship between antecedent conditions at the beginning of each die-off event (SPEI at 24 and 48 month intervals) and its duration and intensity was used to test whether the previous long-term water balance had any effect on the drought attributes during the event using linear regression (at *P* < 0.05). Heat waves were identified as three consecutive days between October and March over the 90th percentile threshold (based on a 15-day analysis window) and were calculated using the “CTX90pct” method outlined in Perkins and Alexander (2012). In their appraisal of a large range of heat-related indices, Perkins and Alexander (2012) showed that the CTX90pct method to calculate heat waves was suitable for capturing trends in heat waves in Australia and produces an adequate population of measurable events that are optimal in describing “extreme” *versus* “measurable” periods of heat stress.

Climate projections were generated for the 15 sites using the CSIRO Mk 3.5 GCM and A2 emissions scenario at a medium sensitivity (WCRP CMIP 3, http://esg.llnl.gov:8443/data/). Intermodel comparisons show that this model produces moderate warming and some of the largest declines in annual rainfall across Australia and performs very well for the key features of the Australian climate (Watterson 2012) (see Fig S4). The requirements of daily GCM data over the analysis period restricted the choice of suitable models for the analysis. Daily data of precipitation and maximum and minimum surface temperature were downscaled to the fifteen sites using two different approaches. Firstly, historic GCM precipitation data (1891–2010) from the nearest grid point were matched to observed precipitation data (1891–2010) using a daily translation method (Mpelasoka and Chiew 2009). Secondly, daily temperatures from the GCM grid cell were scaled to the observed station data using a constant factor (ratio of daily mean GCM historic to observed temperatures) and then applied to the GCM future data. SPEI values, MTI values, and drought event characteristics could then be computed for the period between 1891–2010 (historic) and 1891–2050 (future), based on a continuous monthly time series of precipitation and potential evaporation. A sensitivity analysis was conducted to test for the effect of projected changes in temperature and precipitation independently. Projected changes in temperature and precipitation were applied to the observed station data (1961–2010) using monthly scaling factors for maximum and minimum temperature (°C change per °C of global warming) and precipitation (percentage change per °C of global warming). All analyses were performed using the R program and relevant packages (v2.11.1, R Foundation for Statistical Computing).

## Results

**Figure 1:**
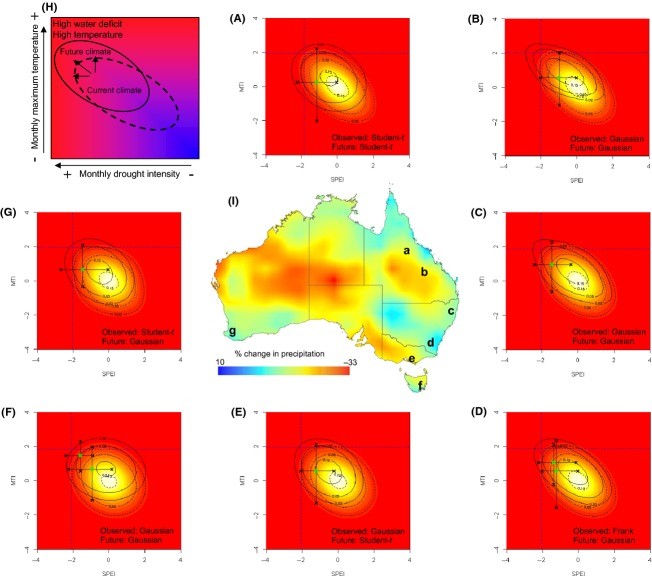
Probability of observed and future changes in drought intensity and maximum temperature. (A–G) Subset of tree die-off sites from a range of vegetation types across Australia showing fitted joint probability density of SPEI and MTI for observed (1891–2010; shaded background and dashed lines) and future projections (2011-2050 based on CSIRO Mk 3.5, A2 SRES scenario; solid contour lines). The three contour lines denote probability densities at 0.15,0.05, and 0.02. The mean SPEI and MTI value (green square) and corresponding maxima and minima (black crosses) during the documented drought-induced die-off events at each site is plotted (see Fig. S1 for an example of how these were derived). Dashed vertical and horizontal lines indicate probabilities of SPEI < 0.02 percentiles and MTI > 0.98 percentiles (for their unimodal distributions) under observed climate conditions. The copula distribution fitted to the observed and future projection is given in the lower right for each panel. The top left hand inset (H) gives an overview for interpreting the joint probability density of monthly drought intensity and maximum temperature at any individual site. The trajectory of future climate is shown by arrows indicating that climate may change in terms of both temperature and/or drought (combination of precipitation and temperature). (I) Map of Australia showing the distribution of projected percentage changes in precipitation for 2050 (using CSIRO Mk 3.5, A2 SRES scenario) based on an observed baseline (1975–2004) and locations of die-off events denoted by lower case letters corresponding to panels A–G.

**Figure 2:**
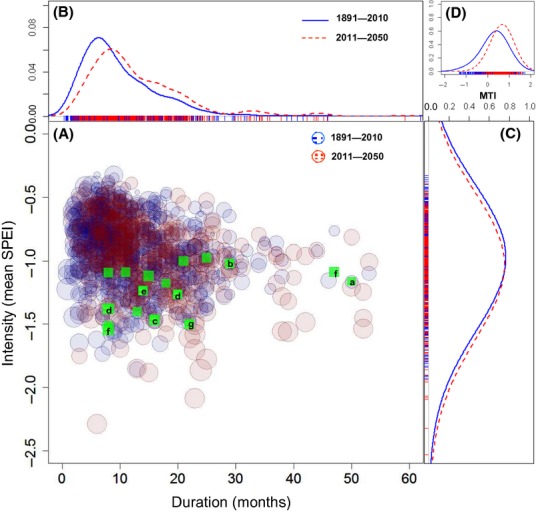
Projected changes in duration and intensity of future drought events relative to the historic climate for 15 tree die-off sites. (A) Drought duration *versus* intensity for observed (1891–2010) and future (2011–2050) datasets (using CSIRO Mk 3.5, A2 SRES scenario). Kernel density estimates of historic (solid blue line) and future (dashed red line) for (B) drought duration, (C) mean drought intensity, and (D) mean maximum temperature intensity (MTI). The mean maximum temperature intensity during each event is given by the size of the circle. The droughts associated with tree die-off events are shown as green squares and lowercase letters correspond to the seven sites shown in Fig. 1A–G.

Using the observed climate record (1891–2012), our analysis showed that 17 die-off events across a range of vegetation types occurred between 1941 and 2012 when conditions were both exceptionally dry and hot (Fig. 1A–G, Table 1). The impact (% of stand affected ranged from ∼7–90%), total extent, and spatial patterning of these events were not consistent across the landscape with die-off occurring along gullies and riparian areas as well as shallow, exposed slopes and hill tops (Table S2). During die-off events, drought duration ranged from 8 to 50 months and mean intensity (mean SPEI) ranged from −1.00 to −1.54 (more negative value represents higher drought intensity), highlighting the range, duration and intensity of conditions capable of inducing die-off (Fig. 2A, Table 1). Among all observed die-off events, the monthly sequence of SPEI values reached probabilities of <0.02 for 1 month or more, regardless of whether the event was of high intensity/low duration or low intensity/high duration (Figs. 1 and 2A, Table 1), suggesting that extreme conditions of this magnitude or greater were required to induce drought die-off.

**Table 1:** Details of the drought attributes associated with documented die-off events in Australia. Drought intensity represents the mean of all monthly SPEI values during the event. The monthly minimum SPEI and maximum MTI values represent extreme water deficit and high temperature conditions during the event, and their corresponding percentiles are given in brackets. Superscript letters after the site name refers to panels shown in Fig. 1.

Site	Start year	Duration (months)	Intensity (mean SPEI)	Min SPEI (min percentile)	Max MTI (max percentile)	No. of heat waves	Predrought SPEI (24 month)
Alpha^b^	2001	46	−1.16	−2.57 (0.007)	2.66 (0.999)	17	−0.55
Armidale^c^	1982	16	−1.47	−2.46 (0.003)	2.29 (0.997)	16	−2.26
Bollon	1979	29	−1.02	−1.86 (0.020)	2.04 (0.993)	14	0.76
Canberra^d^	1965	8	−1.38	−2.24 (0.005)	2.13 (0.987)	4	0.59
Canberra^d^	1981	20	−1.26	−2.05 (0.011)	2.37 (0.998)	16	−0.62
Charters Towers^a^	1991	50	−1.16	−2.26 (0.003)	2.23 (0.995)	25	1.57
Cobar	1965	18	−1.18	−1.82 (0.019)	2.35 (0.997)	4	0.85
Cooma	1965	8	−1.09	−1.83 (0.016)	2.13 (0.988)	4	0.95
Hobart^f^	1977	47	−1.09	−2.27 (0.006)	1.97 (0.988)	7	1.03
Hobart^f^	2012	9	−1.54	−2.55 (0.001)	2.29 (0.998)	3	0.36
Ipswich	1977	15	−1.12	−2.10 (0.011)	1.74 (0.971)	8	0.99
Jarrahdale^g^	2010	22	−1.50	−2.70 (0.001)	2.10 (0.995)	6	1.27
Mt Macedon^e^	1967	14	−1.24	−2.09 (0.010)	2.11 (0.989)	7	0.05
Mathinna	1967	8	−1.51	−3.21 (0.001)	2.37 (0.991)	1	0.44
Yeelirrie	1976	21	−1.00	−1.98 (0.020)	1.74 (0.969)	5	0.91
Tumbarumba	2002	13	−1.40	−2.14 (0.004)	1.80 (0.976)	7	0.99
Wilcannia	1941	11	−1.09	−2.04 (0.017)	1.96 (0.983)	4	0.20
	**Mean**	**22**	**−1.25**	**−2.22 (0.009)**	**2.10 (0.989)**	**9**	**0.44**
	**Min**	**8**	**−1.54**	**−3.21 (0.001)**	**1.39 (0.969)**	**1**	**−2.26**
	**Max**	**50**	**−1.00**	**−1.82 (0.020)**	**2.66 (0.999)**	**25**	**1.57**

For 14 of the 17 die-off events, the MTI values reached probabilities > 0.98 of the observed range during the drought event (Table 1), while all events surpassed MTI probabilities > 0.97. At least one heat wave (see Methods for definition) occurred during each of the documented tree die-off events (Table 1). Among all drought events in the sites' climate records, 99% of “extreme” droughts (see definition below) contained at least one heat wave highlighting the relevance of short stochastic periods of temperature stress to vegetation experiencing water deficit. By analyzing the conditions common to all die-off events, we classified “extreme” droughts as those that included months that were <0.02 percentile for SPEI, >0.98 percentile for MTI and included at least on heat wave event. This definition allows us to distinguish between more common drought events (where SPEI < 0.33 percentile) that occur, on average, 1 in 3 year, from extreme droughts that occur less frequently at 1 in 24 year for the observed climates among the 15 documented sites (Table 2).

**Table 2:** Percentage change in projected or future (2011–2050) drought events relative to historic data (1891–2010) for all die-off sites in the analysis. Historic and future drought attributes are based on drought analysis of daily data from CSIRO Mk 3.5, A2 SRES scenario, medium sensitivity. Drought integral refers to the sum of monthly SPEI values during the event. Extreme events are defined as those with duration ≥8 months and where monthly SPEI and MTI values <0.02 and >0.98 percentiles, respectively. The return interval refers to the mean number of years between drought events.

Site	All drought events					Extreme drought events		
	% change in mean duration	% change mean intensity	% change mean integral	% change frequency per decade	% change heat waves	% change frequency per decade	Historic return interval (years)	Future return interval (years)
Alpha	76	8	107	−12	122	11	14	12
Armidale	3	1	11	19	66	11	18	16
Bollon	11	6	18	48	−55	48	21	8
Canberra	−10	6	−2	36	72	85	18	10
Charters Towers	29	9	35	16	49	11	25	25
Cobar	30	15	47	44	184	122	18	7
Cooma	−16	14	−9	48	82	27	14	12
Hobart	35	2	42	−9	118	−26	41	49
Ipswich	22	1	31	24	90	91	31	8
Jarrahdale	35	6	42	9	114	11	21	16
Mt Macedon	26	0	25	38	101	234	25	8
Mathinna	32	6	41	2	41	178	62	10
Yeelirrie	−15	8	−6	79	117	122	18	7
Tumbarumba	0	8	4	53	65	−36	18	25
Wilcannia	13	15	38	30	98	85	21	10
**Mean**	**18**	**7**	**28**	**28**	**84**	**65**	**24**	**15**
**Min**	**−16**	**0**	**−9**	**−12**	**−55**	**−36**	**14**	**7**
**Max**	**76**	**15**	**107**	**79**	**184**	**234**	**62**	**49**

The influence of the SPEI sequence prior to documented die-off events was examined to assess whether die-off events were consistently associated with a particular set of antecedent conditions (i.e., exceedingly dry or wet conditions). Documented die-off events occurred after both wet and dry periods; in all observed die-off events the 24-month SPEI (at the start of the drought event) ranged from −2.26 to 1.57 (Table 1). Furthermore, the duration and intensity of extreme drought events within the climate record were never correlated with either the 24-or 48-month SPEI based on our regression analyses (data not shown).

The mean monthly SPEI versus MTI values during die-off events were plotted on the fitted joint probability density function of observed SPEI and MTI (Fig. 1A–G). For about one-third of the sites, a likelihood ratio test suggests that the best fit of the joint distributions for the observed climates were Student-*t* or Frank copula distributions. For these sites, the fitted copula distributions indicated strong (Student-*t*) or weak (Frank) tail dependence between the SPEI and MTI values (Fig. 1B,C and E, Table 3). The joint probability density function using precipitation and temperature predictions from a moderate warming and dry scenario were also fitted to show changes in drought conditions (Fig. 1, Table 3). Future climates at all sites showed a trend toward greater probability of hotter and drier drought conditions, and the probability of sites experiencing the threshold corresponding to extreme conditions (defined as SPEI ≤ 0.02 percentile and MTI ≥ 0.98 percentile) increased by 56–270% across the different sites (Table 3).

**Table 3:** Details on the type of model used to fit the joint drought intensity and maximum temperature distribution for the observed and projected or future (based on the CSIRO Mk 3.5, A2 SRES scenario) for each site. For the observed data, the probability of extreme conditions represents the joint probability at the 0.02 and 0.98 percentiles for the observed monthly SPEI and MTI marginal distributions respectively. The future probability of extreme conditions represents the probability under the projected joint distribution function at the 0.02 and 0.98 percentiles of the observed monthly SPEI and MTI marginal distributions respectively. Superscript letters after the site name refers to panels shown in Fig. 1.

Site	Observed		Future		
	Fitted distribution	Joint probability of extreme conditions	Fitted distribution	Joint probability of extreme conditions	% change from observed
Alpha^b^	Gaussian	0.01	Gaussian	0.07	214
Armidale^c^	Gaussian	0.02	Gaussian	0.03	106
Bollon	Gaussian	0.01	Student-*t*	0.03	173
Canberra^d^	Frank	0.02	Gaussian	0.04	56
Charters Towers^a^	Student-*t*	0.02	Student-*t*	0.06	174
Cobar	Gaussian	0.02	Gaussian	0.06	259
Cooma	Gaussian	0.02	Gaussian	0.03	59
Hobart^f^	Gaussian	0.02	Gaussian	0.04	86
Ipswich	Frank	0.03	Gaussian	0.07	135
Jarrahdale^g^	Student-*t*	0.01	Gaussian	0.03	139
Mt Macedon^e^	Gaussian	0.03	Student-*t*	0.08	129
Mathinna	Frank	0.02	Gaussian	0.04	114
Yeelirrie	Gaussian	0.02	Gaussian	0.07	218
Tumbarumba	Gaussian	0.02	Gaussian	0.05	226
Wilcannia	Gaussian	0.02	Gaussian	0.08	270
				**Mean**	**157**
				**Min**	**56**
				**Max**	**270**

Under the relatively moderate warming scenario with associated declines in precipitation used here, drought events became longer, more intense and/or more frequent (Fig. 2A–D, Table 2). In general, the frequency of predicted drought events relative to the historic data increased by an average of 28% (−12 to 79%) across all sites with a 20% and 7% increase in duration and intensity respectively (Table 2). More importantly, the frequency of events considered extreme and capable of inducing tree die-off increased at all sites by 65% (−36 to 234%), changing from occurring at 1 in 24 years to 1 in 15 years (Table 2). The incidence of heat waves also doubled and droughts were more likely to co-occur with heat waves (Table 2). A small number of predicted droughts fell outside the margins of the observed record and are perhaps indicative of “mega-drought” conditions, characterized by higher intensities and longer durations than has been observed in the historic record (Fig. 2A).

**Figure 3:**
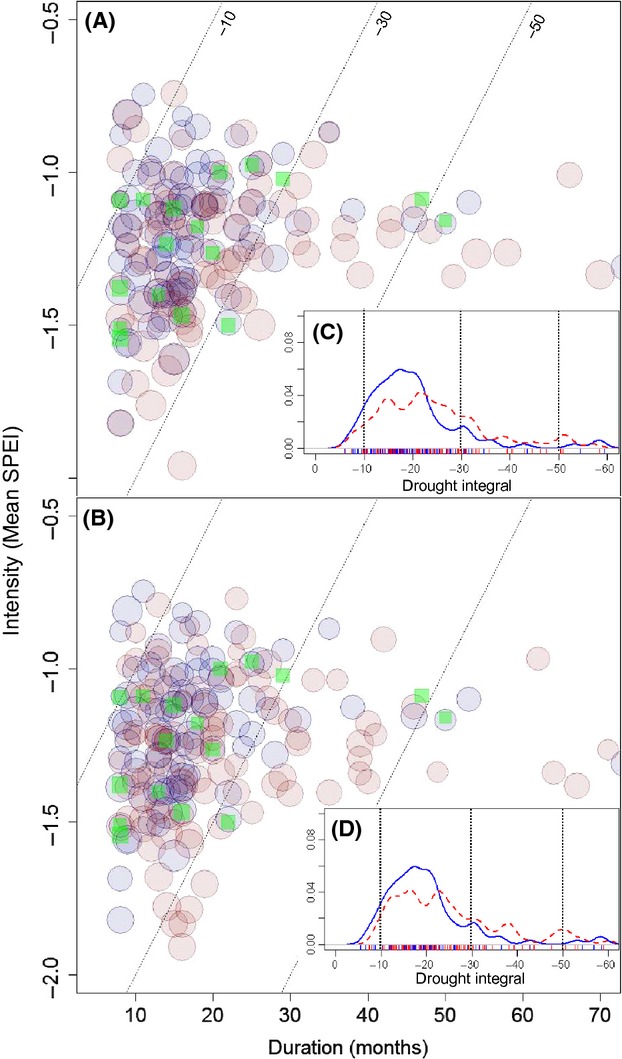
Future changes in temperature or precipitation have an approximately equal effect on drought events. Blue circles on all plots show the relationship between mean drought intensity and duration for drought events (SPEI <0.02 percentile and duration ≥ 8 months for observed climate; 1961–2010). Red circles show simulated climate data perturbed by (A) temperature alone and (B) precipitation only (based on projections at 2050) for the 15 die-off sites (green squares denote drought associated with die-off events). The kernel density estimates of the drought integrals (sum of monthly SPEI values during drought) for all events based on observed (solid blue line) and perturbed climate data (red dashed line) for (C) temperature and (D) precipitation. Dashed reference lines (A–D) denote drought integral values at −10, −30, and −50.

Given that predictions of precipitation are particularly variable across GCMs and scenarios, we performed a sensitivity analysis to help distinguish the role of temperature and precipitation on drought attributes. Simulated changes to observed records (1961–2010) of temperature (∼1.44 °C change from 2010 climate) and precipitation (11–27% annual decline by 2050) were found to increase drought event duration by approximately 22% with little change in mean drought intensity (Fig. 3, Table S5 and S6) relative to the observed record. Changes in the frequency and drought integral (sum of monthly drought SPEI values) for drought events (<0.02 percentile and ≥ 8 months) were also similar for climates with simulated changes in temperature or precipitation (Fig. 3C and D). Our results also show a sharp increase in the frequency of heat wave events (74%) and an increased likelihood of a heat wave occurring during an extreme drought event (mean increase of 113% Table S5) associated with simulated changes in temperature.

## Discussion

Key attributes of climatic drought stress were assessed using a probabilistic approach that allowed us to describe the conditions capable of inducing drought damage and mortality in a consistent manner across a range of vegetation types. Droughts coinciding with the documented die-off events reached minimum SPEI values of less than 2% probability of occurrence within the observed record (Table 1). This finding suggests that regardless of regional climatic differences, tree populations among many species in Australian ecosystems tolerate at least 98% of the climatic conditions they experience and become vulnerable to drought stress events beyond this common climatic threshold. The occurrence of high maximum temperatures (MTI > 0.98 percentile in the vast majority of cases) and heat waves during all of the documented events reinforces the importance of both water and heat stress acting in concert to bring about severe drought damage and die-off. Based on the climate profiles of the documented die-off events, we estimate that conditions that permit extreme droughts occur, on average, every 24 years (ranging from 14 to 62 years across sites). Robust and consistent estimates of drought occurrence provide an important benchmark for understanding the significance of drought in shaping vegetation patterns and modeling future changes in drought impacts.

It is important to stress that these thresholds of drought intensity and high temperatures define an enhanced likelihood of tree die-off (above background rates) for a given site/region experiencing a similar drought history. Once drought events surpass these thresholds, the magnitude of the drought attributes (e.g., duration) and second-order factors such as soil type, topography, species, stand dynamics and disturbance history will affect the extent and pattern of die-off. For example, it is likely that significant portions of the landscape (e.g., mid slope and valley floor) may only exhibit mild changes in vegetation structure and function, that is, reduced leaf area, while more sensitive sites (e.g., upper or exposed slopes) may sustain severe drought damage and tree die-off (Matusick et al. 2013). However, given the opportunistic and inherently biased nature of die-off observations (i.e., times when the presence or absence of die-off is often not reported) and lack of long-term monitoring in most ecosystems, the data used for this analysis cannot provide deterministic predictions of tree die-off for an extreme drought event without a more detailed appraisal of the sensitivity of the system through time and space. Nevertheless, this approach does provide insights into long-term and regional-level patterns in vegetation responses to extremes in water deficit and temperature and provides empirical estimate of likely return intervals for extreme droughts across a broad suite of ecosystems in Australia.

The coincidence of high temperatures and heat waves with severe drought such as those documented here and elsewhere (Williams et al. 2013) is likely to escalate even in the absence of systematic declines in precipitation (Fig. 3). It is well established that summer rainfall and surface air temperature are negatively correlated at global and continental scales (Lyon 2011; Mueller and Seneviratne 2012; Vautard et al. 2007). Thus, heat waves are common during drought, and the combined effect of reduced summer rainfall and heat waves creates greater stress on plants than either factor alone (De Boeck et al. 2011). Average maximum air temperatures were elevated during drought months (Fig. 1A–G, Table 1), but more importantly, short periods of heat stress during summer were likely to have been pivotal in initiating tree die-off and mortality processes, when tree water and carbon status were already diminished (Allen et al. 2010; Galiano et al. 2011). During drought conditions, trees are more vulnerable to heat stress due to the lack of transpiration-mediated cooling of the foliage, enhanced heat loads on tissues, and increased light stress (Barua and Heckathorn 2006). Furthermore, increased evaporative demand will accelerate the decline in tree water status and promote dehydration of plant tissues.

The lack of consistency in drought conditions preceding an observed event suggests that the antecedent meteorological conditions (assessed at 24 and 48 months) may provide limited prediction of the occurrence of die-off, suggesting additional biotic and abiotic factors not considered here are likely to be important for vegetation sensitivity to drought. For example, Fensham (1998) note that the persistence of wet periods over decadal and interdecadal periods can increase vegetation cover in northeastern Australian savannahs, thereby enhancing competition for water and making these communities more vulnerable to die-off during subsequent drought events. The reverse may also be true, whereby extreme drought in the preceding years helps to reduce mortality for subsequent events and help to stabilize the system (Lloret et al. 2012).

This study characterizes the joint probability density function of the two key determinants of climatic stress to provide a realistic assessment of current and future risks to tree survival, across their climatic niche and at the edges of their distribution (Fig. 1). By populating the bioclimatic space with observations of drought impacts and mortality, the limits of plant adaptation to drought can be quantified across a two-dimensional climatic space. While there are some similarities with this approach and other environmental envelope modeling involving associations among a suite of average climate parameters to evaluate species distributions, they are not directly comparable. An important element of our approach was to select climatic indices that have a direct functional role in limiting survival rather than using a suite of climate variables that may only be correlated with species distribution. This means that our analysis is likely to be more robust in defining biologically relevant thresholds for episodic type die-off events. The characteristics of the copula distributions themselves tell us how the likelihood of dry and hot conditions change as droughts become more extreme. The majority of these distributions conformed to the Gaussian or normal multivariate distribution with SPEI and MTI values showing differing levels of covariation. This tail dependence (Student-*t* copula), or lack thereof (Frank copula) in about one-third of the sites, implies that the co-occurrence of hot, dry conditions characteristic of extreme events occurs at proportionally higher likelihood, in the case of the Student-*t* distribution, or lower likelihood, in the case of the Frank distribution likelihoods, than would be predicted for their univariate distributions. For some ecosystems an increased or decreased coincidence of water deficit and high temperatures as part of a changing drought regime, will be a strong selective force for defining species adaptation to extreme drought events. We show that this multivariate approach is necessary for capturing the interactive effects of climatic stress and providing a means to quantify changes in extreme drought conditions that have been derived from multisite analyses. (Fig. 1 and Table 3). This type of approach will help to resolve questions around the extent to which historic and recent changes in extreme conditions are symptomatic of a climatic shift and provide a means to assess changes in the probability of climatic thresholds likely to influence key issues around ecosystem dynamics, for example terrestrial carbon stores (Schwalm et al. 2010).

The analysis establishes the predominant patterns in the observed drought regime within the duration *versus* intensity space and allowed us to assess changes in these attributes into the future (based on a moderate warm and relatively dry scenario) across different regions and vegetation types (Fig. 2). In general, drought intensified under the projected future climate across all sites, yet on a site-by-site basis this intensification tended to be associated with increases in duration or intensity or both (Table 2). Quantifying changes in the key attributes of drought events such as duration and intensity is critical for understanding the mechanisms that underpin differences in responses that occur among individuals, species, and communities during severe droughts (Fensham and Fairfax 2007; Koepke et al. 2010). Our current understanding of mortality mechanisms maintains that plant water and carbon balances are strongly linked to drought intensity by modulating soil and atmospheric water deficit across the soil-to-leaf hydraulic pathway and duration given the rate-limited nature of carbon assimilation and depletion (Mitchell et al. 2013b). Thus, the combination of intensity and duration of an “extreme” type drought, as described here, may have differential impacts on a particular species, population, and/or cohort. While the approach presented here only describes the primary meteorological drivers of droughts capable of inducing tree die-off, these data are crucial inputs for predicting site water balance in more detailed models that describe plant responses to drought events (McDowell et al. 2013). For example, shorter and more intense droughts are likely to kill trees via hydraulic failure, a process that has been widely attributed to episodic die-off events (Anderegg et al. 2012; Martínez-Vilalta and Piñol 2002; Nardini et al. 2013). Alternatively, an increase in long-duration events at similar or reduced intensity may restrict carbon assimilation in species with relatively conservative stomatal regulation and potentially leading to carbohydrate starvation (McDowell 2011; Poyatos et al. 2013). Perhaps more importantly, prolonged droughts and elevated temperatures also increase the risk of biotic attack, while plant's defenses are compromised and/or conditions promote greater activity and expansion of pest populations (Mattson and Haack 1987; Raffa et al. 2008). Changes in the frequency of extreme drought under the scenario presented here (Table 2) and elsewhere (Mpelasoka et al. 2008) may also reduce vegetation resilience through time if a complete recovery of plant vasculature, carbohydrate status and defensive mechanisms is not realized in the intervening years between drought events. A small number of predicted droughts fell outside the margins of the observed record and are perhaps indicative of “mega-drought” conditions, characterized by higher intensities and longer durations than have ever been observed in the historic record (Fig. 2A). The climatic drivers of these mega-drought events under the projections used in this study originated from both increased temperature and altered circulation patterns that create prolonged declines in precipitation. If realized, these climate events may generate unprecedented, extensive die-off that could induce long-term shifts in vegetation structure and function.

Results from the sensitivity analysis of drought to changes in precipitation and temperature demonstrate that moderate increases in temperature alone have the potential to significantly lengthen drought events and increase the risk of extreme events even in the absence of reductions in precipitation. From a physiological perspective, elevated temperatures not only accelerate dehydration by increasing water loss but can impinge on plant carbon balance as well. Water-stressed plants with significantly reduced carbon assimilation may deplete stored carbohydrates more rapidly when temperatures are elevated (Adams et al. 2013). In turn, this may reduce plant fitness and impair recovery following drought, as carbohydrates are required for new growth during recovery from drought (Brodribb et al. 2010; Galiano et al. 2011). Changes in carbon metabolism in response to elevated temperatures would have significant effects on ecosystem carbon budgets, by increasing respiration rates and the duration over which the ecosystem remains a potential source of atmospheric CO_2_ (Zhao and Running 2010; Keith et al. 2012).

This study shows that the climatic drivers of episodic tree die-off across vegetation types and biomes in Australia can be characterized using a common drought surface and that tree die-off occurs beyond common thresholds in these indices. This study suggests that plant functioning adapts to variability in climate and the prevailing drought regime over a similar range of conditions. If this pattern were consistent globally, it would lend support to the concept that woody species from both wet and dry environments are similarly vulnerable to changes in water availability and temperature (Choat et al. 2012). The data presented in this study suggest that “top-down” approaches can yield important data on climatic thresholds of dominant species in their respective ecosystems, can be readily coupled to models of ecosystem dynamics from the local to global scale, and used to assess future mortality risk in forest systems. The attributes of drought events inducing less severe impacts such as changes in net primary productivity and recruitment can also be assessed under this approach, thereby providing a means to characterize drought consequences on a range of ecosystem processes. Although the impact of extreme events such as drought may not always invoke a response in a plant community of similar extremity (Smith 2011), the nature of changes in drought conditions shown here may be rapid and severe enough to have significant and lasting impacts on species distributions and ecosystem dynamics. The phenotypic and genotypic plasticity of tree species, and associated stand-level responses to shifts in water availability, are the key determinants of future changes in community composition and distribution. If the thresholds associated with tree survival are relatively fixed, as suggested by physiological evidence surrounding hydraulic failure (Martínez-Vilalta and Piñol 2002), then the likelihood of drought events crossing these thresholds and inducing mortality will increase significantly under future climate scenarios for many forest and woodland ecosystems globally.

## Supporting Information

**Figure S1.** Drought history and details of the 2010–2011 drought event near Jarrahdale in Australia's south west. **Figure S2.** Comparison of four GCMs and three scenarios for different sites across Australia. **Table S1.** Details of the 15 documented die-off sites used in this study (sorted by climate wetness; precipitation divided by potential evaporation). **Table S2.** Statistical parameter estimates from the joint distribution selection routine including; the log likelihood ratio, tau and degrees of freedom. **Table S3.** Percentage change in drought attributes between observed data perturbed by temperature change (based on CSIRO Mk 3.5, A2 SRES scenario, ∼1.44°C change from 2010 climate) relative to observed data (1961–2010) for all documented die-off sites. **Table S4.** Percentage change in drought attributes between observed data perturbed by precipitation change (based on CSIRO Mk 3.5, A2 SRES scenario) relative to observed data (1961–2010) for all documented die-off sites.
