# Supplementary material for: Exposure of trees to drought-induced die-off is defined by a common climatic threshold across different vegetation types
Source: Ecol Evol. 2014 Mar 6;4(7):1088–101. doi: 10.1002/ece3.1008 (PMC3997324; doi:10.1002/ece3.1008)
Supplement: Supplementary file 1 — Figure S1. Drought history and details of the 2010–2011 drought event near Jarrahdale in Australia's south west. Figure S2. Comparison of four GCMs and three scenarios for different sites across Australia. Table S1. Details of the 15 documented die-off sites used in this study (sorted by climate wetness; precipitation divided by potential evaporation). Table S2. Statistical parameter estimates from the joint distribution selection routine including; the log likelihood ratio, tau and degrees of freedom. Table S3. Percentage change in drought attributes between observed data perturbed by temperature change (based on CSIRO Mk 3.5, A2 SRES scenario, ∼1.44°C change from 2010 climate) relative to observed data (1961–2010) for all documented die-off sites. Table S4. Percentage change in drought attributes between observed data perturbed by precipitation change (based on CSIRO Mk 3.5, A2 SRES scenario) relative to observed data (1961–2010) for all documented die-off sites. [file ece30004-1088-sd1.pdf]

Supporting Information

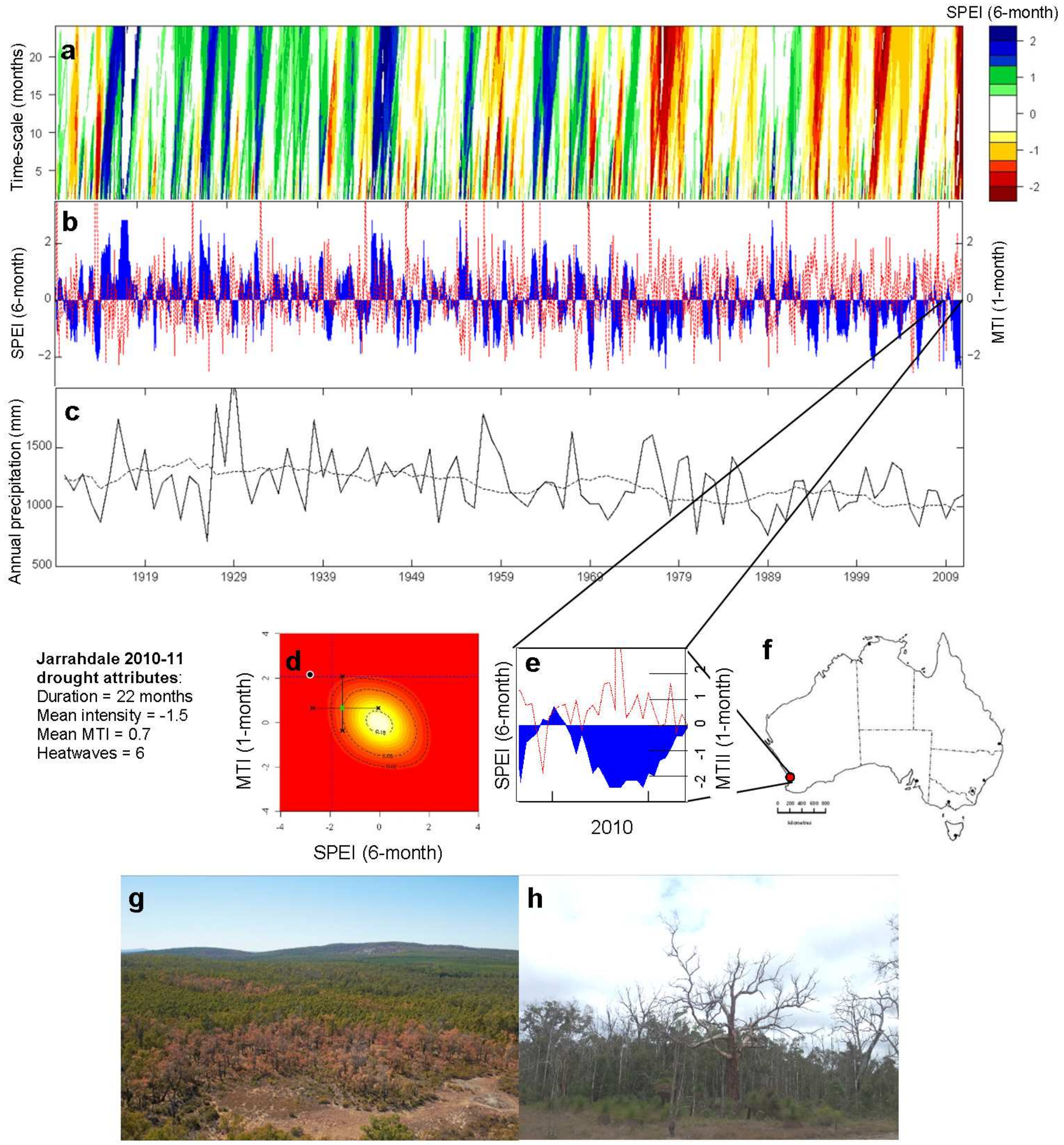

Figure S1. Drought history and details of the 2010-2011 drought event near Jarrahdale in Australia's south west. Time-series plots (1910 – 2011) of a) SPEI values at multiple time scales (1 to 24 month) with values corresponding to colours given on right hand side, b) SPEI (6-month time-scale, blue filled line) and maximum temperature intensity (red dashed line) and c) annual precipitation (solid line) and 10 year moving average annual precipitation (dashed line). d) Joint probability density (represented with a Student-*t* copula with 11 degrees of freedom) of SPEI and MTI values with mean SPEI and MTI value (green square) and minima and maxima (black crosses) from the 2010 –11 drought. e) Magnified subset of the SPEI (blue filled line) and MTI (red line) plot during the 2010-11

drought event. f) Location of mortality site within Australia. g) aerial photograph and h) ground photograph of tree mortality in the Jarrah forest where mortality occurred in stands of *Eucalyptus marginata* and *E. calophylla* on predominately shallow soils near rocky outcrops such as those depicted here.

Table S1. Details of the 15 documented die-off sites used in this study (sorted by climate wetness; precipitation divided by potential evaporation).

Site #	Site ID	Location	Lat/long	Timing	Mortality event type	Climate MAP, MAT and P/PET[a]	Biome[8]	Forest type	Species affected	Symptoms	% of stand affected and spatial pattern of die-off	Magnitude of tree die-off/ patch size	Additional factors/biotic agents	References
1	**Yeelirrie**	Yeelirrie, WA	-27°17', 120°15'	1976-78	Episodic mortality	240, 20.5, 0.08	Deserts and Xeric Shrublands	Mulga shrubalnd	*Acacia aneura*	Not reported	11 %, diffuse across the stands			(Fox, 1980)
2	**Wilcannia**	Wilcannia, NSW	-31° 41', 142°43'	1944-45	Long-term and episodic mortality	258, 19.4, 0.13	Temperate Grasslands, Savannas and Shrublands	Mulga shrubalnd	*A. aneura, Flindersia maculosa*	Not reported	~ 90 % within affected stands, occurring in a 'face' in thick patches of trees and more isolated death in scattered stands.	~10'000 km^2/not reported	Scarab beetle found not thought to be the cause	(Condon, 1949)
3	**Cobar**	Cobar, Western NSW	-31°36', 145°50'	1965-66	Episodic mortality	381, 18.9, 0.17	Temperate Grasslands, Savannas and Shrublands	Mulga shrubalnd	*A. aneura, Callitris columellaris,E. populnea, Eremophila sturtii, Alectryon oleifolius*	Not reported – repeated sampling confirmed deaths	40 %, not reported	Not reported	Not reported	(Cunningham and Walker, 1973)
4	**Alpha**	Central Queensland	-23°28', 146°21'	2001-05	Episodic mortality	564, 21.9, 0.25	Tropical and Subtropical Grasslands, Savannas and Shrublands	Savanna woodland	*E. melanophloia, E. populnea, C. clarksoniana* and *C. dallachiana*	Recently dead identified as bark still firm and weak epicormic growth	~15 %,tree death was greater at sites with higher clay content	Not reported	Not reported	(Fensham and Fairfax, 2007)
5	**Bollon**	Mungalla Ck, QLD	-28° 02', 147° 2'	1979-1980	Episodic mortality	464, 21.6, 0.27	Temperate Grasslands, Savannas and Shrublands	Open woodland	*E. camaldulensis*	Total leaf fall	22 %, riparian areas close to river bed	~1000 km^2/10-100 ha	Not reported	(Gordon et al. 1988)
6	**Charters Toweres**	Great Basalt Wall, QLD	-20°00', 145°20'	1992-1994	Episodic mortality	667, 23.5, 0.33	Tropical and	Savanna woodland	*E. xanthaclada, Corymbia*	Not reported	27 %, grazed (cattle and macropod)	Not reported/10-	Not reported	(Fensham, 1998)

							Subtropical Grasslands, Savannas and Shrublands		*erythrophloia* and *C. dallachiana*		areas on deeply weathered basalt	100ha		
**7**	**Canberra**	Canberra and Hall, ACT	-35° 16', 151° 38'	1965 and 1982-1983	Episodic mortality	598, 13.3, 0.39	Temperate broadleaf and mixed forest	Sclerophyll forest, woodland	*E. rossii, E. macroryncha, E. albens, E. cinerea, Casuarina cunnighamiana, E. blackelyi, E. bridgesiana, E. melliodora, E. dives, Amyema pandula* (mistletoe)	Foliage wilted and browned off, bark shrinkage and fissuring and ultimately separation at the cambium	29 %, exposed rocky slopes and shallow soils, ridge tops	Not reported/10-100 ha	Infestation of wood-boring and defoliating insects	(Pook and Forrester, 1984); (Landsberg 1985)
**8**	**Cooma**	Wambrook, NSW	-36° 10', 149° 00'	1965	Episodic mortality	515, 11.4, 0.43	Temperate broadleaf and mixed forest	sub-alpine and alpine heath, sub-alpine woodland	Observed in different locations - sub alpine heath, *Kunzea muelleri* and *Podocarpus lawrenci; E. rossii-E. polyanthemos; Callitris endlicheri and Casuarina stricta; E. rubida, E. vimnalis, E. puciflora* and *E. stellulata*	Foliage wilted and browned off	~ 10 %,shallow soils and rocky outcrops	Not reported/10-100 ha	Infestation of wood-boring and defoliating insects	(Pook et al. 1966)
**9**	**Ipswich**	Pullenvale and Moggill, QLD	-27° 33', 152° 50'	1977-1978	Long-term decline	849, 16.6, 0.52	Temperate broadleaf and mixed forest	Woodland (disturbed grazing land)	E. tetricornis, E. maculata, E. tessellaris, E. propinqua, E. acmenoides	Epicormic resprouting	Not reported, floodplain sites	Not reported/1-10 ha	Leaf tunnelling and skeletonising insects	(Landsberg and Wylie 1983)
**10**	**Hobart**	Hobart, TAS	-42° 55', 147° 17'	1977-1981 and 2012-2013	Episodic mortality	645, 11.4, 0.61	Temperate broadleaf and mixed forest	Dry sclerophyll forest (mixed species)	*E. pulchella, E. viminalis, E. globulus*. Includes both understorey and	Leaf loss	8 %, shallow soils (< 0.2 m)on hill slopes	Not reported/1-10 ha	None reported	(Kirkpatrick and Marks 1985) and *unpublished data*

									overstorey spp.					
11	**Armidale**	New England Tablelands, NSW	-30° 25′, -149° 05′	1982-1983	Long-term decline	775, 13.6, 0.62	Temperate broadleaf and mixed forest	Open woodland	*Eucalyptus laevopenea, E. blackelyi*	Dieback affected - Vigorous epicormic shoots	13 %, most severe on poorly drained valley floor, spreading to slopes later	5000km^2/10-1000 ha	Heavy infestation by defoliating insects	(Crombie and Milburn 1985); (Mackay et al., 1984)
12	**Mt Macedon**	Mt Towrong, VIC	-37° 25′, 144° 38′	1967-68,	Episodic mortality	815, 11.5, 0.74	Temperate broadleaf and mixed forest	Sclerophyll forest	*E. geniocalyx, E. obliqua*	Total browning of foliage	30 %, shallow soils on hill slopes	Not reported/1-10 ha	None reported	(Ashton and Spalding 2001),
13	**Jarrahdale**	Darling scarp/SW WA	-32°19′, 116° 04′	2010-2011	Episodic mortality	1161,16.6, 0.75	Mediterranean Forests, Woodlands and Scrub	Dry sclerophyll forest/woodland	*E. marginata, E. bullich*	Browning of foliage and branch shedding	26 % within stands on shallow soils on rocky outcrops	~10000 km^2/1-10 ha	None detected	(Matusick et al., 2012)
14	**Mathinna**	Fingal, Tasmania	-41° 32', 147° 59′	1967	Episodic mortality	834, 11.5, 0.84	Temperate broadleaf and mixed forest	Dry sclerophyll forest (mixed species)	Predominately death and dieback of *E. obliqua*. To a lesser extent *E. viminalis* (but not as severe) and Very occasional death of *E. sieberi*	Not reported	~20 %,gully /Riparian forest - generally undisturbed with little road or logging occurring	Not reported/1-10 ha	Some infestation with leaf skeletonizers but not found to be the cause	(Felton 1972), *unpublished data*
15	**Tumbarumba**	Tumbarumba, NSW	-35°39′ 148°09′	2002-03	Episodic mortality	969, 13.0, 0.78	Temperate broadleaf and mixed forest	Temperate eucalypt forest	*E. delegetensis, E. dalrympleana, E. stellulata*	Not reported - repeated sampling confirmed deaths	~ 7 %, patchy, trees died on gullies when seeps ceased to flow	500 km^2/Not reported	Canopy attacked by psyllid insects causing significant reductions in leaf area	(Keith et al. 2011)

[a] MAP is the mean annual precipitation, MAT is the mean annual temperature and P/PET is the ratio of precipitation to potential evaporation

* Biome classification based on the Interim Biogeographic Regionalisation for Australia version 7 (2012)

References for observed die-off sites

1. Fox JED (1979) Stability in Mulga stands in times of drought. *Annual Report, Mulga Research Centre* 3:23-28.
2. Condon, R W. 1949. "Mulga Death in the West Darling Country." *Journal of the Soil Conservation Service of New South Wales* 38: 7–14.
3. Cunningham GM & Walker PJ (1973) Growth and survival of Mulga (Acacia aneura F. Muell. Ex benth) in Western New South Wales. *Tropical Grasslands* 7(1):69-77.
4. Fensham RJ & Fairfax RJ (2007) Drought-related tree death of savanna eucalypts: Species susceptibility, soil conditions and root architecture. *Journal of Vegetation Science* 18(1):71-80.
5. Gordon G, Brown aS, & Pulsford T (1988) A koala (*Phascolarctos cinereus* Goldfuss) population crash during drought and heatwave conditions in south-western Queensland. *Austral Ecol* 13(4):451-461.
6. Fensham RJ (1998) The influence of cattle grazing on tree mortality after drought in savanna woodland in north Queensland. *Aust J Ecol* 23(4):405-407.
7. Pook EW, Costin AB, & Moore CWE (1966) Water stress in native vegetation during the drought of 1965. *Aust J Bot* 14:257-267; Pook, E.W. and R. Forrester. (1984). Factors influencing dieback and drought-affected dry sclerophyll forest tree species. Australian Forestry Research. 14:201-217; Landsberg J. (1985) Drought & dieback of rural eucalypts. *Australian Journal of Ecology*, **10**, 87-90.
8. Pook EW, Costin AB, & Moore CWE (1966) Water stress in native vegetation during the drought of 1965. *Aust J Bot* 14:257-267
9. Landsberg J & Wylie FR (1983) Water stress, leaf nutrients and defoliation, a model of dieback in rural eucalypts. *Aust J Ecol* 8:27-41.
10. Kirkpatrick JB & Marks F (1985) Observations on drought damage to some native plant species in eucalypt forests and woodlands near Hobart, Tasmania. *Pap Proc R Soc Tasman* 119:15-21; O'Grady (*unpublished data*).
11. White, T.C.R. (1986) Weather, Eucalyptus dieback in New England, and a general hypothesis of the cause of dieback. *Pacific Science* 40, 58-78; Crombie SD & Milburn JA (1985) Water Relations of Rural Eucalypt Dieback. *Aust J Bot* 36:233-237. Mackay SM, Humphreys FR, Clark RV, Nicholson DW, Lind PR (1984) Native Tree Dieback and Mortality on The New England Tablelands of New South Wales. In: *Research Paper No. 3.* pp 1-23, Forestry Commission of N.S.W.
12. Ashton DH, Bond H, Morris GC (1975) Drought damage on Mount Towrong, Victoria. *Proceedings of the Linnean Society of New South Wales,* **100**, 44 – 69; Ashton DH, Spalding DK (2001) The

ecology of a stressful site : Mount Towrong , Central Victoria 1967-1997. *Australian Forestry*, 143-150.

13. Matusick G., Ruthrof K., Brouwers N., Dell B. & Hardy G.J. (2013) Sudden forest canopy collapse corresponding with extreme drought and heat in a mediterranean-type eucalypt forest in southwestern Australia. *European Journal of Forest Research*, 132, 497-510.
14. Felton (1972) Eucalypt diebacks in Tasmania. *Appita* 26:207-208.
15. Keith H, van Gorsel E, Jacobsen KL, & Cleugh Ha (2011) Dynamics of carbon exchange in a Eucalyptus forest in response to interacting disturbance factors. *Agricultural and Forest Meteorology*.

Table S2. Statistical parameter estimates from the joint distribution selection routine including; the log likelihood ratio, tau and degrees of freedom. The Gaussian and Frank copulas do not have a degrees of freedom estimate.

Site	Copula – joint distribution	Log likelihood ratio value	Tau	Degrees of freedom	Log likelihood	Tau	Degrees of freedom
			Observed			Future	
**Alpha**	Student-t	190.01	-0.47	217.78	190.04	-0.48	217.78
**Alpha**	Gaussian	**190.39**	**-0.48**		**104.44**	**-0.55**	
**Alpha**	Frank	170.44	-3.03		93.43	-3.65	
**Armidale**	Student-t	128.75	-0.40	156.01	190.01	-0.48	196.88
**Armidale**	Gaussian	**129.14**	**-0.40**		**97.64**	**-0.53**	
**Armidale**	Frank	116.20	-2.48		88.73	-3.59	
**Bollon**	Student-t	146.59	-0.43	34.85	**109.34**	**-0.56**	**11.14**
**Bollon**	Gaussian	**146.11**	**-0.43**		106.12	-0.55	
**Bollon**	Frank	145.06	-2.82		104.79	-3.93	
**Canberra**	Student-t	131.91	-0.41	93.70	131.91	-0.41	93.70
**Canberra**	Gaussian	132.11	-0.41		**103.51**	**-0.54**	
**Canberra**	Frank	**134.09**	**-2.69**		99.24	-3.75	
**Charters Towers**	Student-t	**167.72**	**-0.45**	**29.17**	14.88	-0.22	94.18
**Charters Towers**	Gaussian	166.72	-0.45		**14.98**	**-0.22**	
**Charters Towers**	Frank	153.46	-2.90		8.35	-0.99	
**Cobar**	Student-t	146.84	-0.43	94.02	146.90	-0.43	127.52
**Cobar**	Gaussian	**147.01**	**-0.43**		**93.39**	**-0.52**	
**Cobar**	Frank	138.03	-2.73		84.50	-3.40	
**Cooma**	Student-t	52.78	-0.26	142.29	52.78	-0.26	142.29
**Cooma**	Gaussian	**53.06**	**-0.26**		**77.00**	**-0.48**	
**Cooma**	Frank	52.81	-1.62		70.52	-3.09	
**Hobart**	Student-t	23.32	-0.17	116.85	23.32	-0.18	116.85
**Hobart**	Gaussian	**23.34**	**-0.18**		**3.49**	**-0.11**	
**Hobart**	Frank	20.81	-1.02		2.96	-0.59	
**Ipswich**	Student-t	83.14	-0.33	37.82	83.14	-0.33	27.82
**Ipswich**	Gaussian	82.02	-0.33		**106.73**	**-0.55**	
**Ipswich**	Frank	**85.95**	**-2.12**		99.65	-3.77	
**Jarrahdale**	Student-t	**44.77**	**-0.23**	**10.96**	24.99	-0.28	43.82
**Jarrahdale**	Gaussian	38.98	-0.23		**24.84**	**-0.28**	
**Jarrahdale**	Frank	39.77	-1.43		19.31	-1.54	
**Mt Macedon**	Student-t	61.89	-0.29	137.81	**29.53**	**-0.30**	**11.01**
**Mt Macedon**	Gaussian	**62.23**	**-0.29**		27.75	-0.30	
**Mt Macedon**	Frank	53.09	-1.63		26.32	-1.85	
**Mathinna**	Student-t	36.40	-0.22	206.35	21.51	-0.27	116.38
**Mathinna**	Gaussian	36.78	-0.22		**21.79**	**-0.68**	
**Mathinna**	Frank	**37.26**	**-1.36**		21.51	-1.63	
**Yeelirrie**	Student-t	58.22	-0.28	131.42	66.84	-0.45	107.90
**Yeelirrie**	Gaussian	**58.50**	**-0.28**		**66.92**	**-0.45**	

**Yeelirrie**	Frank	54.91	-1.66		59.76	-2.78	
**Tumbarumba**	Student-t	58.37	-0.28	130.86	84.32	-0.50	150.07
**Tumbarumba**	Gaussian	**58.64**	**-0.28**		**84.47**	**-0.50**	
**Tumbarumba**	Frank	55.18	-1.67		78.07	-3.22	
**Wilcannia**	Student-t	104.26	-0.36	100.87	61.73	-0.44	94.42
**Wilcannia**	Gaussian	**104.49**	**-0.37**		**61.75**	**-0.43**	
**Wilcannia**	Frank	96.54	-2.24		58.48	-2.77	

Figure S2. Comparison of four GCMs and three scenarios for different sites across Australia. Monthly scaling factors at 2050 for maximum temperature, minimum temperature and precipitation are given for four models covering the expected range (CSIRO Mk3.5, GFDL-21, MIROC-M and MRI-GCM232) of future climates over Australia. Lines are means of three SRES scenarios (A2, A1FI and B1) and shaded regions representing ± 1 standard error. The model and scenario used in this study (CSIRO Mk3.5, A2 at 2050) is shown in solid black line for comparison.

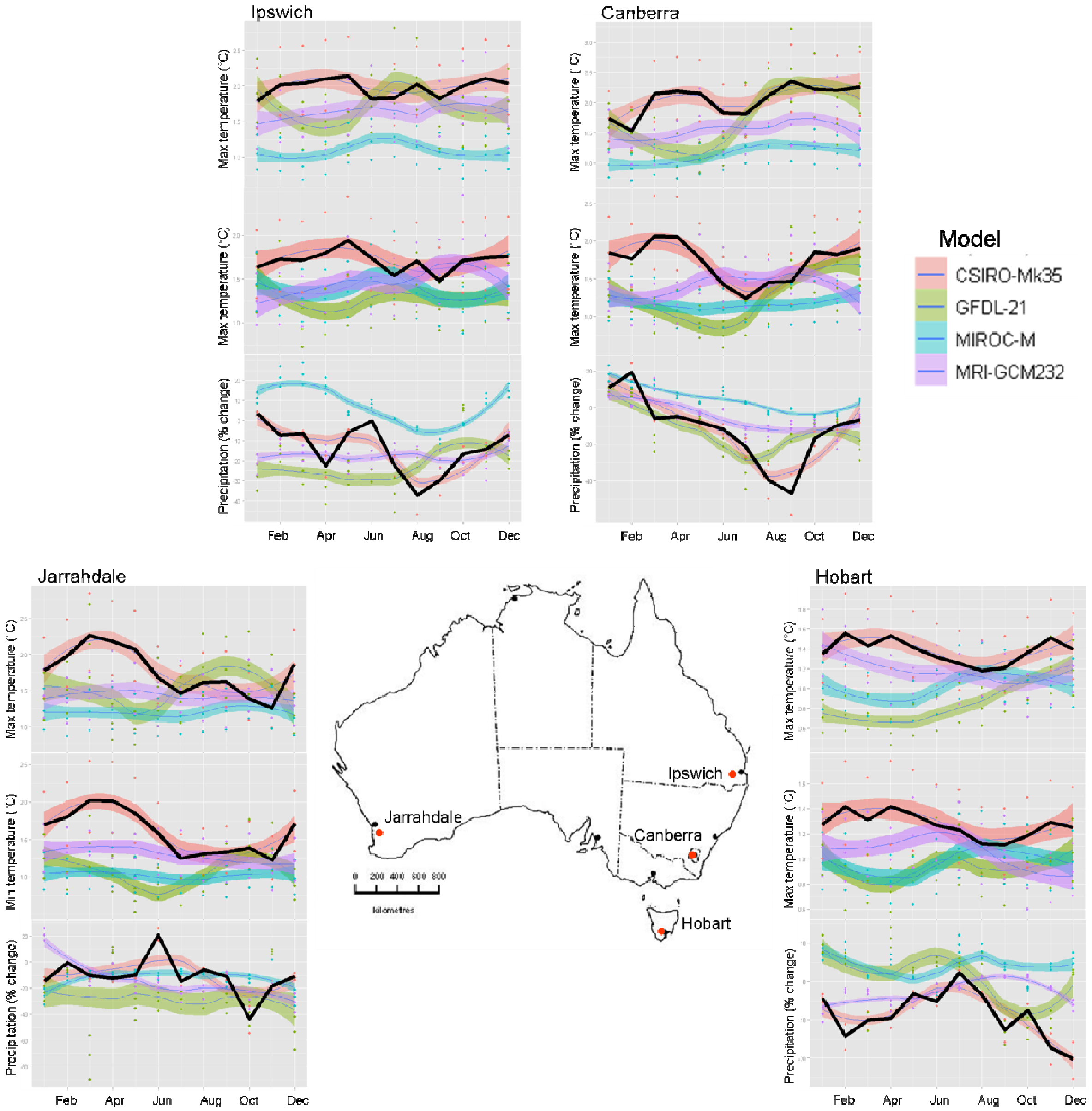

Table S3. Percentage change in drought attributes between observed data perturbed by temperature change (based on CSIRO Mk 3.5, A2 SRES scenario,~1.44 °C change from 2010 climate) relative to observed data (1961-2010) for all documented die-off sites. Mean intensity is the mean SPEI-six month for all months experiencing drought.

				Frequency per decade			
**Site**	**Duration (months per**	**Mean duration**	**Mean intensity**	**All events**	**Extreme events***	**Heat waves**	**Extreme event coinciding with a heat wave**
**Alpha**	6	23	5	-14	-40	12	0
**Armidale**	40	46	2	-4	40	96	102
**Bollon**	0	0	0	0	0	0	0
**Canberra**	14	33	5	-14	20	34	107
**Charters Towers**	5	10	8	-5	100	33	152
**Cobar**	27	21	-9	5	-33	69	100
**Cooma**	24	-1	5	25	-17	43	17
**Hobart**	4	13	6	-8	50	29	180
**Ipswich**	12	39	14	-19	25	62	107
**Jarrahdale**	17	62	2	-28	17	269	369
**Mt Macedon**	13	42	2	-20	20	45	87
**Mathinna**	21	10	-2	10	25	80	170
**Yeelirrie**	27	38	-1	-8	14	251	251
**Tumbarumba**	11	5	0	6	0	40	27
**Wilcannia**	0	0	0	0	-20	50	31
**mean**	**15**	**23**	**2**	**-5**	**13**	**74**	**113**
**min**	0	-1	-9	-28	-40	0	0
**max**	40	62	14	25	100	269	369

* Extreme events are defined as those with duration ≥ 8 months and where monthly intensity reached SPEI values < 0.02 percentile.

Table S4. Percentage change in drought attributes between observed data perturbed by precipitation change (based on CSIRO Mk 3.5, A2 SRES scenario) relative to observed data (1961-2010) for all documented die-off sites. The observed values are given in brackets. Mean intensity is the mean SPEI-six month for all months experiencing drought. Frequencies of events are expressed as events per decade.

				Frequency per decade	
	**Duration (months per decade)**	**Mean duration**	**Mean intensity**	**All events**	**Extreme events***
**Alpha**	26	26	-1	0	-20
**Armidale**	31	26	8	4	80
**Bollon**	18	3	5	15	33
**Canberra**	22	12	-3	10	40
**Charters Towers**	15	35	7	-15	100
**Cobar**	34	28	-9	5	-33
**Cooma**	23	7	2	15	0
**Hobart**	5	14	9	-8	50
**Ipswich**	11	37	13	-19	25
**Jarrahdale**	53	23	23	24	133
**Mt Macedon**	19	48	6	-20	0
**Mathinna**	27	27	-1	0	0
**Yeelirrie**	83	29	14	42	129
**Tumbarumba**	16	4	4	11	25
**Wilcannia**	4	-9	-7	14	-20
**Mean**	**25**	**21**	**4**	**5**	**36**
**min**	4	-9	-9	-20	-33
**max**	83	48	23	42	133

* Extreme events are defined as those with duration ≥ 8 months and where monthly intensity reached SPEI values < 0.02 percentile.
